# Supplementary material for: Conditional chemoconnectomics (cCCTomics) as a strategy for efficient and conditional targeting of chemical transmission
Source: eLife. 2024 Apr 30;12:RP91927. doi: 10.7554/eLife.91927 (PMC11060718; doi:10.7554/eLife.91927)
Supplement: Figure 3—source data 3. [file elife-91927-fig3-data3.zip › Figure 3 source data3 part 1.zip!/Figure 3-Source data 3-part1/1067-9-CRISPResso_Report_c33kf3u3.zip!/Nucleotide_percentage_quilt_around_sgRNA_AGCCAGGTGTCGCAGACGAT.pdf]

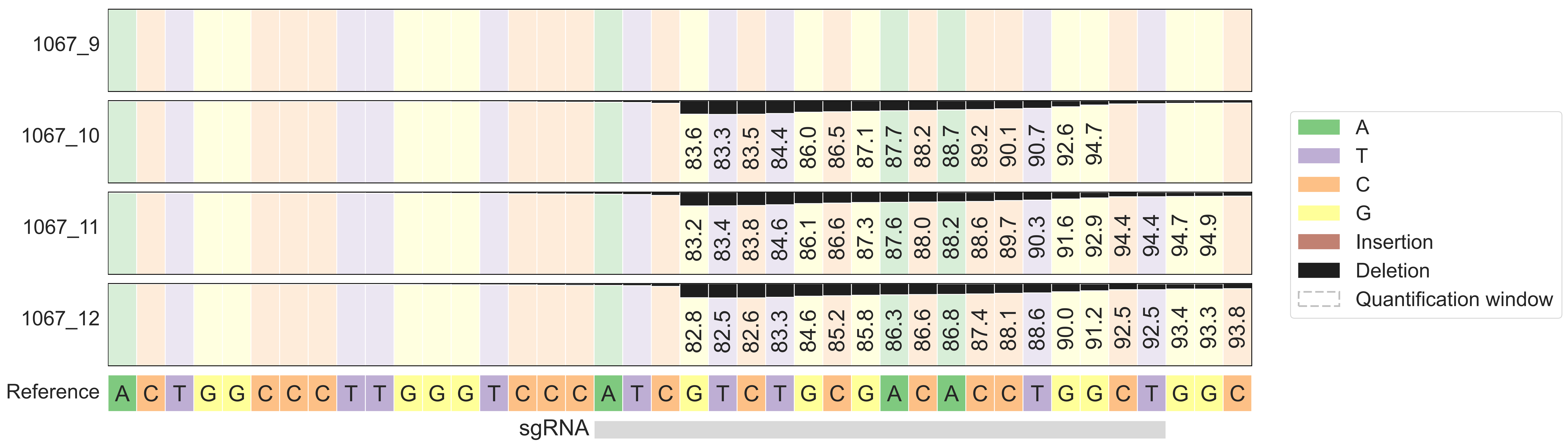
1067_9
1067_10
83.6 83.3 83.5 84.4 86.0 86.5 87.1 87.7 88.2 88.7 89.2 90.1 90.7 92.6 94.7
1067_11
83.2 83.4 83.8 84.6 86.1 86.6 87.3 87.6 88.0 88.2 88.6 89.7 90.3 91.6 92.9 94.4 94.4 94.7 94.9
1067_12
82.8 82.5 82.6 83.3 84.6 85.2 85.8 86.3 86.6 86.8 87.4 88.1 88.6 90.0 91.2 92.5 92.5 93.4 93.3 93.8
Reference
A C T G G C C C T T G G G T C C C A T C G T C T G C G A C A C C T G G C T G G C
sgRNA
A
T
C
G
Insertion
Deletion
Quantification window
